# Platelet-Rich Plasma and Micrografts Enriched with Autologous Human Follicle Mesenchymal Stem Cells Improve Hair Re-Growth in Androgenetic Alopecia. Biomolecular Pathway Analysis and Clinical Evaluation

**DOI:** 10.3390/biomedicines7020027

**Published:** 2019-04-08

**Authors:** Pietro Gentile, Maria G. Scioli, Alessandra Bielli, Barbara De Angelis, Ciro De Sio, Domenico De Fazio, Gabriele Ceccarelli, Angelo Trivisonno, Augusto Orlandi, Valerio Cervelli, Simone Garcovich

**Affiliations:** 1 Department of Surgical Sciences, Plastic and Reconstructive Surgery, University of Rome “Tor Vergata”, 00173 Rome, Italy; bdeangelisdoc@gmail.com (B.D.A.); valeriocervelli@virgilio.it (V.C.); 2 Department of Biomedicine and Prevention, Institute of Anatomic Pathology, University of Rome “Tor Vergata”, 00173 Rome, Italy; scioli@med.uniroma2.it (M.G.S.); alessandrabielli@hotmail.it (A.B.); orlandi@uniroma2.it (A.O.); 3 Private Plastic Surgeon, 00168 Rome, Italy; dr.cdesio@gmail.com (C.D.S.); defazioplastic@gmail.com (D.D.F.); dott.a.trivisonno@gmail.com (A.T.); 4 Department of Public Health, Experimental Medicine and Forensic, Human Anatomy Unit, University of Pavia, 27100 Pavia, Italy; gabriele.ceccarelli@unipv.it; 5 Center for Health Technologies, University of Pavia, 27100 Pavia, Italy; 6 Institute of Dermatology, F. Policlinico Gemelli, IRCCS, Università Cattolica del Sacro Cuore, 00168 Rome, Italy; simgarko@yahoo.it

**Keywords:** micrografts, HF-MSCs, human follicle mesenchymal stem cells, PRP, platelet rich plasma, hair loss, hair-regrowth, PRP hair, stem cells hair

## Abstract

Platelet rich plasma (PRP) and Micrografts containing human follicle mesenchymal stem cells (HF-MSCs) were tried as a potential treatment for androgenetic alopecia (AGA). However, little to no work has yet to be seen wherein the bio-molecular pathway of HF-MSCs or PRP treatments were analyzed. The aims of this work are to report the clinical effectiveness of HF-MSCs and platelet-rich plasma evaluating and reviewing the most updated information related to the bio-molecular pathway. Twenty-one patients were treated with HF-MSCs injections and 57 patients were treated with A-PRP. The Wnt pathway and Platelet derived-growth factors effects were analyzed. 23 weeks after the last treatment with mean hair thickness increments (29 ± 5.0%) over baseline values for the targeted area. 12 weeks after the last injection with A-PRP mean hair count and hair density (31 ± 2%) increases significantly over baseline values. The increment of Wnt signaling in Dermal Papilla Cells evidently is one of the principal factors that enhances hair growth. Signaling from mesenchymal stem cells and platelet derived growth factors positively influences hair growth through cellular proliferation to prolong the anagen phase (FGF-7), inducing cell growth (ERK activation), stimulating hair follicle development (β-catenin), and suppressing apoptotic cues (Bcl-2 release and Akt activation).

## 1. Introduction

A clinical need exists for the development of biotechnologies to improve the hair re-growth in androgenetic alopecia (AGA).

AGA is a dynamic and chronic hair loss disorder, affecting 80% of white men and 40% of women before age 70, in which lymphocytes and mast cells have been seen around the miniaturizing follicle detailed in the stem cell-rich lump zone [1,2,3,4]. Miniaturization of the follicles is characterized by a diminishment of anagen phase, with an improvement in the amount of resting hair follicles, telogen, containing microscopic hairs in a hairless scalp [5,6,7]

Current treatments affirmed for AGA include drugs as Finasteride, topical lotions such as Minoxidil, and surgery as hair transplantation [2]. In hair loss scalp, hair follicle stem cell numbers stay unaltered, though the number of more actively proliferating progenitor cells particularly diminishes [8].

In this way, the aim of hair-tissue engineering (HTE) must be the development of new autologous-technologies to involve hair re-growth by in vitro and ex vivo culture or by in vivo regeneration and bio-stimulation. Autologous stem cells have been of great interest for application in hair-regrowth. Some early efforts in the field focused on isolating primary cells from a biopsy of the tissue of interest and growing the cells ex vivo for subsequent introduction back into the patient. 

In the last year (2017), the authors reported the results of a medical device called Rigeneracons^®^ (CE certified Class I, Human Brain Wave, Turin, Italy) to provide autologous micro-grafts enriched of human follicle mesenchymal stem cells (HF-MSCs) immediately available to be used in patients affected by AGA. The micrografts were obtained by the disaggregation of a 2 mm punch biopsy with the selection of a cell population with a diameter of 50 microns. High cell viability was reported. However, a major limitation encountered in this area has been the difficulty in expanding cells to sufficient numbers for human use, the necessity to perform this expansion in Good Manufacturing Practices (GMP) laboratories, and the viability of the expanded cells [9].

For this reason, the clinical use of HF-MSCs to improve hair re-growth has not been adequately considered.

In addition, the use of autologous platelets derived growth factors can represent valid support in hair-tissue regeneration (HTR) for their capacity to promote cell proliferation, differentiation and neo-angiogenesis, favoring the wound healing process [10,11,12]. In fact, platelet-rich plasma (PRP) contains at least six major growth factors, including basic fibroblast growth factor (bFGF), platelet-derived growth factor (PDGF), vascular endothelial growth factor (VEGF), epidermal growth factor (EGF), transforming growth factor-β (TGF-β), and insulin-like growth factor-1 (IGF-1) released after platelet activation [11]. Each one of these major growth factors is involved in a specific bio-molecular activity.

The authors intended to clear up the clinical and bio-molecular impacts of HF-MSCs and PRP scalp infusion in people affected by AGA describing the bio-molecular pathway suggested in hair-regrowth. Information reviewed and reported exhibit the clinical efficacy and histological safety of the PRP and HF-MSCs treatments.

In this work, in particular, the recent advances of hair-tissue engineering using HF-MSCs, PRP, and new biotechnologies, were discussed reporting the most innovative results achieved, and the possible drawbacks or risks associated with the clinical translation of these technologies.

The examination convention agreed to the Declaration of Helsinki, the European and Italian rules. All patients gave written informed consent before partaking in the investigation.

## 2. Experimental Section

### 2.1. Rules and EMA/CAT Recommendations

This retrospective observational case-series study was conducted following the principles outlined in the Declaration of Helsinki and internationally consented ethics in clinical research [13]. A quality assessment was carried out based on the Strengthening the Reporting of Observational studies in Epidemiology (STROBE) checklist [14].

The protocol was conducted in strict adherence to the European Rules represented by Regulation n.1394/2007 of the European Parliament (EC) and by the Reflection Paper on characterization of cutting edge treatment medicinal products draft concurred, 20 June 2014 EMA/CAT/600280/2010 Rev 1, Committee for Advanced treatments (CAT), in which the autologous use in one step surgery, minimal manipulation, omofunctional utilization "utilized for an indistinguishable fundamental capacity in the beneficiary as in the donor", manipulation with gadgets in aseptic conditions, are conditions that do not require Good Manufacturing Practices (GMP) rules for processing, Good Clinical Practices (GCP) for the clinical application and the Ethical Committee endorsement. PRP preparation must be performed respecting in Italy “Decree of the Blood, 2 November 2015”, dispositions related to quality and safety parameters of blood and emocomponents. All patients gave written informed consent.

The study protocol, object of a research contract (D.R. 1467/2017) and an university master’s degree called “Regenerative surgery and medicine in wound care management”, was approved with Rectoral Degree (D.R. n. 1794/2018) of 19 September 2018 and the Ethics on Research Committee of the School of Medicine, “Tor Vergata” University, Rome, Italy, with registration number #0031036/2018.

All patients received detailed oral and written information about the study, including the risks, benefits and alternative therapies, and signed an informed consent form before any study procedures.

### 2.2. Bio-Molecular Pathway of Stem Cells and Growth Factors that Improve Hair Re-Growth

- HGF and HGF activator (secreted by DPC): Improve proliferation of follicular epithelial cells;
- EGF: Improves migration and growth of follicle ORS cells by activation of Wnt/β-catenin signaling;
- b-FGF: Improves the development of hairs’ follicles;
- IL-6: Involved in WIHN through STAT3 activation;
- VEGF: Improves peri-follicular angiogenesis;
- TGF-β: Stimulates the signaling pathways that regulate hair cycle;
- IGF-1: Improves survival, migration, and proliferation of hair follicle cells;
- IGFBP-1 to -6: Regulates IGF-1 effects and its interaction with extracellular matrix proteins at the hair follicle level;
- BMP: Maintains DPC phenotype (crucial for stimulation of hair follicle stem cell);
- BMPR1a: Maintains the proper identity of DPCs (essential for specific DPC function);
- M-CSF: Involved in wound-induced hair re-growth;
- M-CSFR: Involved in wound-induced hair re-growth;
- PDGF and PDGFR-β/-α64: Up-regulate the genes involved in hair follicle differentiation. Induction and regulation of anagen. PDGF and its receptors are essential for follicular development;
- Wnt3a: Involved in hair follicle development through β-catenin signaling;
- PGE2: Stimulates anagen in hair follicles;
- PGF2α: and analogs improve the transition from telogen to anagen;
- BIO (GSK-3 inhibitor);
- PGE2 or inhibition of PGD2 or PGD2 receptor D2/ GPR4477: Improve follicle regeneration;
- Iron and l-lysine (Under investigation)

### 2.3. Clinical Study Overview and Patients

**Figure 1:**
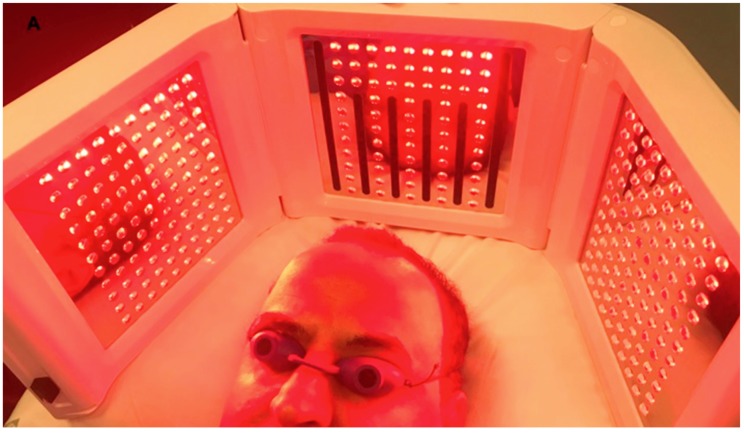
A non-smoker 58-year-old male patient classified androgenetic alopecia (AGA) 3V according to Norwood–Hamilton Scale. During Low level led therapy treatment performed by Geno-Led.

Two works (HF-MSCs and A-PRP) were conducted and reviewed by the authors. This investigation enlisted 78 patients, 56 males who showed AGA in stage II–V as controlled by the Norwood–Hamilton classification scale and 22 females with AGA in stage I–II as dictated by the Ludwig classification scale. Twenty-one patients were treated with HF-MSCs injections and 57 patients were treated with A-PRP. Fundamental and local prohibition criteria were considered. Fundamental expulsion criteria included immunosuppression and cancer, sepsis, and also the utilization of pharmacological therapeutics targeting on AGA (finasteride, similar drugs, and/or antiandrogens) in the earlier year. Localized expulsion criteria included the utilization of topical medicines for AGA in the earlier year. Low-level led therapy (LLLT) was proposed by the authors 15 days after each treatment to stimulate hair regrowth during the HF-MSCs and PRP treatment and every 3 weeks after the treatment until 6months post-treatment (Figure 1). The histological outcomes for patients treated with HF-MSCs and PRP were published by the authors. The Wnt pathway and platelet-derived-growth factors effects were analyzed and reviewed. AGA diagnoses were established on parameters reported in the previous works [3,4,15,16,17].

#### 2.3.1. Preparation of Autologous Micro-Grafts Suspension

**Figure 2:**
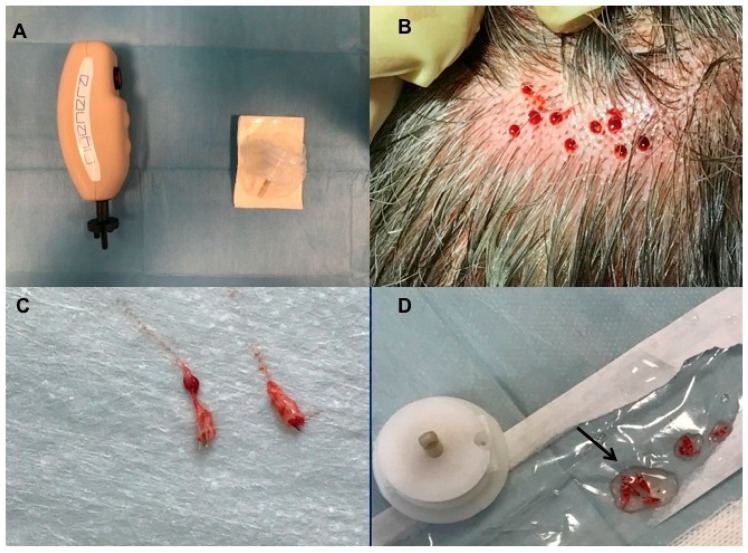
Micro-graft procedure phase 1. (**A**) On the left, Rigenera Securdrill device and on the right Rigeneracons kit; (**B**) The holes in the scalp after punch biopsy; (**C**) Selected scalp tissues into the strips (2 × 2 mm); (**D**) The authors controlled the presence of bulb in the selected tissue and conserved the strips into saline solution.

**Figure 3:**
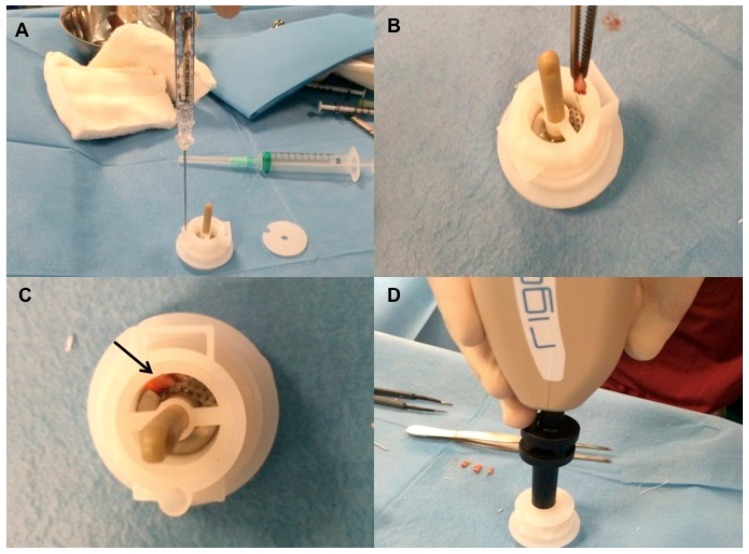
Rigenera procedure phase 2 (positioning of scalp tissue in Rigeneracons and fragmentation by centrifugation). (**A**) The addition of 1.2 mL of physiologic solution into Rigeneracons kit; (**B**) the strips collected into Rigeneracons; (**C**) Deatil of Rigeneracons containing one strip indicated by arrow; (**D**) centrifugation at 80 rpm with Rigenera Securdrill device for 60 s.

Autologous micro-grafts of HF-MSCs for human application were prepared using Rigeneracons (Medical Device with CE certified Class I, product by HBW Srl; Turin, Italy) (Figure 2A) in different steps. First step: harvesting of the scalp with punch biopsy (Figure 2B) and cutting the scalp tissues into the strips (2 × 2 mm) (Figure 2C,D); second step: collecting and disaggregation of the strips under sterile conditions (vertical laminar flow hood) performed by Rigeneracons (Figure 3B,C) in 1.2 mL of saline (NaCl 0.9%) (Figure 3A) through centrifugation at 80 rpm (Figure 3D) with the aim to select cells with a size of 50 μm; third step: collecting the micrograft’s suspension from the system and mechanically infiltration, using 1 mL syringes into the selected area of the patient’s scalp affected by AGA. Samples of micrograft’s solutions obtained were previous cultured and subsequently characterized by cytospin and immunocytochemistry to identify the Human Follicle Stem Cells (HFSCs) (data published). The authors considered HFSCs the cellular population containing HF-MSCs and human hair follicle epithelial stem cells (HF-ESCs).

#### 2.3.2. A-PRP Preparation and Delivery

**Figure 4:**
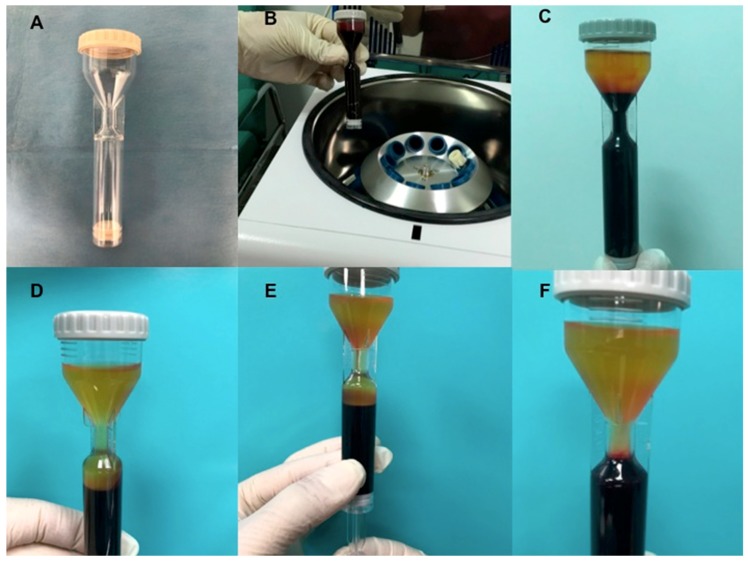
i-Stem platelet rich plasma (PRP) procedure. (**A**) Kit i-Stem; (**B**) The blood collected in i-Stem Kit underwent at centrifugation; (**C**) The PRP and PPP suspension obtained by the system after the first centrifugation; (**D**) The PRP and PPP after the second centrifugation.; (**E**) selection of PRP; (**F**) PRP concentration in the middle side of kit.

Autologous blood (17.7 mL) was harvested using the i-Stem Kit PRP Preparation System (Figure 4A,B) (i-Stem, Biostems, Co., LTD., Seoul, South Korea 138-843, Medical Device, CE and Food and Drug Administration (FDA)) under the approval of the transfusional service. Sodium citrate (ACD) as an anticoagulant, was added (2.2 mL). 

After the first spin (centrifugation at 3000 rpm for 6 min) (Figure 4C), the authors removed the Platelet-Poor Plasma PPP portion (1 mL) and RBC (Red blood cells) (2 mL) and re-centrifuged for the second time (3000 rpm for 3 min) and at the end of the procedure, 15 mL of A-PRP was obtained (Figure 4D–F). Microscopic platelet counts were performed on the A-PRP collected from all participants.

#### 2.3.3. Mechanical and Controlled Injection (MCI) of Autologous Suspension and Study Design

In the patients treated with PRP or with HF-MSCs, the scalp was separated into six regions (as previous published). The interfollicular infusions, (0.2 mL × cm^2^) were realized to targeted regions at a depth of 5 mm, with MCI procedure [17] utilizing an Ultim gun (Anti-Aging Medical Systems, Montrodat, France) outfitted with a 1 mL Luer lock syringe with needle 30-gauge, in two sessions spaced two months apart (relatively to HF-MSCs) and in three session spaced 30 days average using 10 mL Luer-look syringe (relatively to PRP).

Suspension infusions were conveyed to the frontal area while placebo infusions (i.e., saline NaCl 0.9%) were infused in the parietal areas in patients with hair loss confined to the frontal-parietal areas. In like manner, for hair loss restricted to the parietal-vertex regions, the suspension was infused in the parietal area, and saline was infused in the vertex area. Equivalent quantities of autologous micrograft’s suspension and placebo infusions were made.

#### 2.3.4. Evaluation of Hair Re-Growth

**Figure 5:**
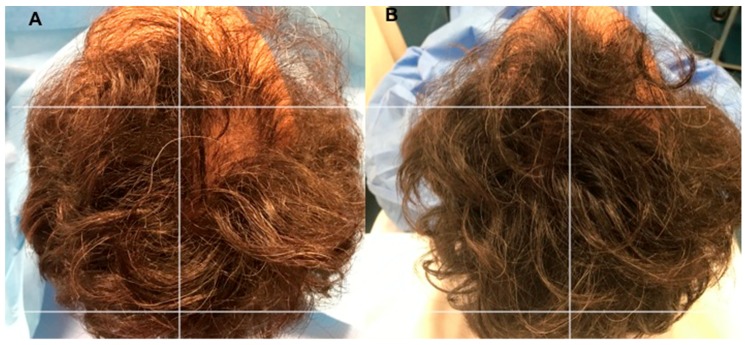
A non-smoker 34-year-old male patient classified AGA 2 according to Norwood–Hamilton Scale. (**A**) Timing T0 (before the treatment) with hair loss localized to the vertex, parietal, temporal, and frontal areas; (**B**) Timing T3 after 12 weeks later the last third treatment with increase of hair density in the right parietal area treated with three autologous platelet-rich plasma not activated (A-PRP) injections versus left parietal area treated with placebo.

**Figure 6:**
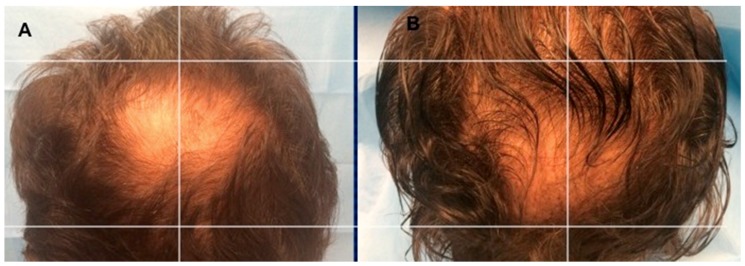
A non-smoker 54-year-old male patient classified AGA 4 according to Norwood–Hamilton Scale. (**A**) Timing T0 (before the treatment) with hair loss localized to the vertex; (**B**) Timing T5 after 23 weeks later the last second treatment with increase of hair density in the vertex area treated with two micrograft injections versus parietal area treated with placebo.

Assessment of hair growth was assessed in five phases: T0, before the first infusion (Figure 5A and Figure 6A); T1 in 3 weeks; T2, in 9 weeks; T3, in 12 weeks (Figure 5B); T4, in 16 weeks, T5 in 23 weeks (Figure 6B), after the last treatment. The hair re-growth assessed after the last treatment was contrasted by picture and the baseline assessment made before medications and between the micrografts and/or PRP treatment region and the manipulation region, which got placebo infusions. The second infusion was performed following two months in the case of HF-MSCs injection. On the other hand, in the case of PRP treatment, the last infusion was the third and each injection was performed each 30 days average.

Photos of the area treated with a suspension of PRP are shown in Figure 5A,B. Photos of the area treated with a suspension of micro-grafts are shown in Figure 6A,B. The impacts of micrografts’ and PRP’s suspension and placebo medications on hair re-growth were evaluated in all patients with the assistance of worldwide picture, doctor’s and patient’s worldwide evaluation scale.

In all patients, two translational regions of hair loss, one at the fringe of the treatment half and a moment along the outskirt of the placebo half, were demarcated with a semi-permanent tattoo.

#### 2.3.5. Statistical Analysis

Values as the mean in addition to baseline mistake were examined by means of Student’s *t*-test, and contrasts considered statistically noteworthy were *p* < 0.05.

## 3. Results

### 3.1. PRP Clinical Results

As beforehand detailed [4,17], 12 weeks later the treatment with A-PRP mean hair count increases significantly over baseline values. In particular, the values obtained, reflect a 31 ± 2% increase in hair density for the treatment group and less than a 1% increase in hair density for the placebo group. Moreover, both the hair count and hair density parameters represent statistically significant improvements in hair growth for the A-PRP treated scalp (Figure 5B) over the placebo-treated control group.

As reported in our previous work [3,4] both patient populations treated with A-PRP (autologous-PRP not activated) and AA-PRP (autologous activated-PRP) respectively, showed an improvement in the number of follicular bulge cells and follicles, epidermal thickening, improved vascularization, and a higher number of Ki67^+^ basal keratinocytes in PRP-treated scalp tissue compared with placebo.

Indeed, histological examination of A-PRP and AA-PRP treated scalp from our previous work [3,4] provides such clinical evidence. 

Hair re-growth at the clinical level showed a similarly positive response to treatment with A-PRP, with patients manifesting significant improvements in hair count and total hair density in the targeted area over the control area (treated with Placebo). Differences between 12 weeks follow-up counts and baseline count for these hair growth parameters were higher in the A-PRP treatment population in this study than in the AA-PRP treatment population in the previous trial performed by the authors [3]. In particular, 3 months hair density measurements for patients treated with A-PRP and AA-PRP were 65 ± 5 and 28 ± 4 hairs/cm^2^, respectively (Table 1 and Table 2). The results obtained constitute a 31 ± 2% increase in hair density when A-PRP treatment is performed versus 19 ± 3% increase in hair density when AA-PRP treatment is performed, with a statistically significant difference in hair growth (*p* = 0.0029) (Table 1 and Table 2). The increase of hair growth parameters for A-PRP over AA-PRP may reflect the greater efficiency of in vivo thrombin to activate platelets and the body to distribute the contents of activated platelets compared with in vitro calcium activation and injection. Moreover, delivery of A-PRP may enable the production of thromboxane A2 (TXA2) by the platelets once they are activated in vivo, which would activate additional platelets and amplify platelet aggregation [18].

### 3.2. Micrografts Clinical Results

12 weeks later the treatment with micro-grafts, mean hair density was increased significantly over baseline values. In particular, the values obtained, reflect a 30 ± 5.0% increase in hair density for the treatment group and less than a 1% increase in hair density for the placebo. Hair Density measurement for area treated with micro-grafts 12 weeks after the last treatment was 39 ± 5 hairs/cm^2^ (Table 1).

As beforehand detailed [15], 23 weeks later the last treatment with micrografts containing HFSCs mean hair density increments (29 ± 5.0%) over baseline values for the treated region and less than a 1% increment in hair density for the placebo region (Figure 6B). At the baseline, no statistical contrasts in hair tally or hair thickness existed between the micrografts treatment region and manipulation region of the scalp.

In the preparatory investigation [15], the authors have built up another strategy to disengage human grown-up stem cells by minimal manipulation based on centrifugation of strips of human hair follicles without expansion or culture. They reported the counting of these cells and the preparatory outcomes acquired by the human follicle stem cells infusions in the scalp of patients affected by AGA, enhancing hair thickness.

Specifically, in the past work, the authors detailed the amount (in term of percentage) of CD44^+^ cells (hair follicle-derived mesenchymal stem cells), from dermal papilla, and the percentage of CD200^+^ cells (hair follicle epithelial-stem cells), from the bulge, gotten via programmed centrifugation of 11 punch tests [15].

The authors revealed, the microscopic assessment performed by cytospin, immunocytochemistry, histological examination got by Hematoxylin-Eosin-stained and clinical assessment, feeling the need to talk about as follow, current advances in the distinctive methodologies to improve regeneration of hair follicles, with accentuation on those including neogenesis of hair follicles in grown-up people utilizing isolated cells and biotechnologies.

## 4. Discussion

It is of extraordinary enthusiasm to discover distinctive methods planning to improve regeneration of the hair follicle under conditions appropriate of a grown-up person. In light of the current knowledge on the epithelial and dermal cells and their associations amid the embryonic hair generation and grown-up hair cycling, numerous researchers have endeavored to acquire mature hair follicles utilizing distinctive methods and methodologies relying upon the reasons for AGA [19,20,21].

Investigations were performed utilizing rat cells, especially from embryonic or infant origin. Notwithstanding, no successful methodology to generate human hair follicles from grown-up cells has yet been accounted for. Maybe the most critical challenge is to give three-dimensional culture conditions mirroring the structure of living tissue. Enhancing culture conditions that permit the extension of particular cells while securing their inductive properties, and additionally, the strategies for choosing populaces of epithelial stem cells should give us the fundamental tools to overcome the challenges that constrain human hair follicle neogenesis [21].

These cells give off an impression of being situated in the lump region of human hair follicles.

Hair follicles are known to contain a well-characterized niche for grown-up stem cells: the lump, which contains epithelial and melanocytic stem cells [22]. Stem cells in the hair lump, an obviously differentiated structure inside the lower permanent portion of hair follicles, can generate the interfollicular epidermis, hair follicle structures, and sebaceous glands [7,23]. The lump epithelial stem cells can likewise reconstitute in a simulated in vivo framework to a new hair follicle [24,25].

Yu et al. [22] demonstrated that follicles of human hairs contain a stem cell populace that can be separated into the smooth muscle cell, neuron and melanocyte heredities in induction medium. Their information demonstrates that Oct4^+^ cells are available in the skin of a human, and the majority of them are situated in the hair follicles in vivo. Oct4 has a place with the family of POU-domain transcription factors that are regularly communicated in pluripotent cells of the developing embryo and mediate pluripotency [26]. Subsequently, follicles of human hair contain multipotent stem cells other than epithelial and melanocytic stem cells, and these cells are situated in the lump region. These cells indicate promising plasticity in ex vivo and in vitro conditions, making them potential candidates for cell engineering and cell substitution treatments.

Each mature hair follicle is a regenerating framework, which physiologically experiences cycles of growth (anagen), relapse (catagen), and rest (telogen) various times in grown-up’s life [27]. In catagen, hair follicle stem cells are kept up in the lump. At that point, the resting follicle re-enters anagen (regeneration) when legitimate molecular signals are given. Amid late telogen to early anagen change, signals from the Dermal Papilla (DP) stimulate the hair germ and quiescent lump stem cells to wind up activated [28]. Numerous paracrine factors are engaged with this crosstalk at various hair cycle stages and some signaling pathways have been implicated [29,30,31]. In anagen, stem cells in the lump offer ascent to hair germs, at that point the transient increasing cells in the grid of the new follicle proliferate quickly to frame another hair filament [32]. As a matter of fact, the authors feel the need to better know which stage is critical to act.

Regeneration of hair follicles was likewise seen in people [33] when dermal sheath tissue was utilized, which was adequate to regenerate additionally the DP structure. After implantation, the whisker DP was equipped for promoting hair follicle regeneration holding the data to decide hair fiber type and follicle size [34].

In an examination reported by Balañá ME et al. [21] the authors prepared in a research facility a dermal-epidermal skin substitute by seeding an a-cellular dermal grid with cultured hair follicle epithelial stem cells and dermal papilla cells (DPCs), both gotten from the grown-up human scalp. These constructs were grafted into a full-thickness wound produced on bare mice skin. In fourteen days, histological structures reminiscent of a wide range of phases of embryonic hair follicle improvement were seen in the grafted region. These structures demonstrated concentric cellular layers of human origin and expressed k6hf, keratin present in epithelial cells of the companion layer. Despite the fact that the presence of completely mature hair follicles was not observed, these outcomes demonstrated that both epithelial and dermal cultured cells from the grown-up human scalp in a dermal scaffold could create in vivo structures that reiterate embryonic hair improvement.

In a recent report published in 2017 by Kalabusheva et al [35], the authors combined postnatal human DPCs and skin epidermal keratinocytes (KCs) in a hanging drop culture to build up a simulated hair follicle germ. The technique depends on DP cell hair-inciting properties and KC self-association. They assessed two protocols of total collecting. Blended HF germ-like structures showed the initiation of epithelial-mesenchymal collaboration, including WNT pathway enactment and expression of follicular markers. They examined the impact of conceivable DP cell niche components including dissolvable components and extracellular matrix (ECM) molecules during the time spent on the organoid assembling and growth. Their outcomes showed that soluble components had little effect on HF germ generation and Ki67^+^ cell score inside the organoids despite the fact that BMP6 and VD3 kept up effectively the DP character in the monolayer culture. Aggrecan, biglycan, fibronectin, and hyaluronic acid (HA) significantly stimulated cell proliferation in DP cell monolayer culture with no impact on DP cell character. A large portion of ECM compounds restricted the growth of cell totals while HA advanced the formation of bigger organoids.

Talavera-Adame et al [36], revealed in a recent study the bio-molecular pathway involved in a cellular treatment. Specifically, it has been additionally demonstrated that Wnt/β-catenin signaling is fundamental for the growth and upkeep of DPCs [37,38]. The increment of Wnt signaling in DPCs evidently is one of the principal factors that enhance hair re-growth [37].

In particular, in a study published by Pirastu N. et al [39], androgen receptor signaling, is implicated by seven genes at six loci. Three main groups were found: genes linked to Wnt signaling (RSPO2; LGR4; WNT10A; WNT3; DKK2; SOX13; TWIST2; TWIST1; IQGAP1; and PRKD1), genes involved in apoptosis (DFFA; BCL2; IRF4; TOP1; and MAPT) and a third more heterogeneous group including the androgen’s receptor and TGF-β pathways (RUNX3; RUNX2; ALPL; PTHLH; RUNX1; AR; SRD5A2; PDGFA; PAX3; and FGF5). Although many different pathways have been implicated in the development of androgenetic alopecia, their results suggest that in addition to the androgen receptor pathway, for which they confirm a prominent function, the Wnt and apoptosis pathways play a fundamental role. Androgenetic Alopecia is characterized by a shorter growth (anagen), which has been associated with increased apoptosis of the hair follicle cells. This result suggests the anagen phase becomes shorter because of differences in the genes regulating apoptosis. The Wnt pathway has been implicated in the transition from the telogen (resting) to the anagen (growth), and also in the determination of the fate of the stem cells in the hair bulge, which are both dysregulated in balding tissue. Finally, baldness risk loci in the WNT ligand biogenesis and trafficking and Class B/2 (Secretin family receptors) pathways were also associated with height, despite none of the individual loci in these pathways being significant: this suggests a “pathway-wide” effect. Therefore, baldness shows pathway-specific genetic correlations, which provide a potential biological basis to observed epidemiological correlations. Pathway-specific genetic correlations hold promise in disentangling the shared biological pathways underpinning complex diseases [39].

Another fascinating field is the likelihood to utilize the fat graft and stromal vascular fraction cells (SVFs) in hair re-growth. SVFs is a heterogeneous group of non-cultured cells that can be dependably removed from fat by utilizing computerized frameworks, in light of centrifugation, filtration and purification of fat tissue or utilizing enzymatic absorption (not proposed by EMA-CAT recommendations). These cells work to a great extent by paracrine systems to help adipocyte viability. Festa et al [40] detailed that adipocyte ancestry cells bolster the stem cell niche and help drive the complex hair growth cycle. This follicular regenerative approach is fascinating and raises the likelihood that one can drive or reestablish the hair cycle in male and female hair loss by stimulating the niche with autologous fat improved with SVF.

Along these lines, Perez-Meza D et al. [41], detailed the safety, tolerability, and quantitative, in patients affected by hereditary alopecia treated with sub-cutaneous scalp infusion of advanced fat tissue. An increment of 31 hairs/cm^2^ was recorded in patients experiencing treatment of fat blended with SVF; one subject who had fat alone reported a mean increment of 14 hairs/cm^2^, proposing that while fat alone may represent an approach for early hairlessness, the addition of SVF may improve this reaction [41]. The discoveries propose that scalp stem cell-enriched fat grafting may represent a promising elective way to deal with treating hair loss in people. Fukuoka et al. [42] inspected the impacts of fat-derived stem cell-conditioned medium infusion in a group of 22 patients affected by AGA. Patients got treatment each 3 to 5 weeks for a total of 6 sessions. The mean increment in the hair count was 29 ± 4.1 in men and 15.6 ± 4.2 in women. No noteworthy distinction was seen amongst men and ladies.

The examination of the investigations reviewed could prompt the conclusion that hair follicle neogenesis utilizing human epithelial and dermal cells is an extremely cumbersome assignment that could require unique culture conditions, some way or another reproducing the ordinary or embryonic skin condition, and the utilization of embryonic or neonatal cells.

In the other hand, the number of papers published on PRP is considerable, but the results are often contradictory. The authors thinking that It is not correct to speak of PRP in general, but it is better to identify different types of PRP preparations depending on their cell content and fibrin architecture. On this way it is possible to identify:

1. Leukocyte-poor PRP (LP-PRP) or Pure Platelet-Rich Plasma (P-PRP). Suspension without leukocytes and with a low-density fibrin network after activation;

2. PRP and Leukocyte (L-PRP). Suspensions with leukocytes and a low densities fibrin network after activation (the largest number of commercial kit);

3. Leukocyte- poor platelet-rich fibrin (LP-PRF) or pure platelet-rich fibrin (P-PRF). Suspensions without leukocytes and a high-density fibrin network.

4. Leukocytes and platelet rich fibrin (L-PRF) or second generations PRP products are preparations with leukocytes and a high-density fibrin network.

As reported, there are too many protocols for preparation of PRP depending on the different time and RPM used, the number of platelets, the availability of growth factors and chemokines. There is also a wide biological (between patients) and temporal (day to day) variation [43]. So, it is difficult to assess which kit for PRP preparation is better and which is worse [44].

Different PRP products might be more or less appropriate to treat different types of tissues and pathologies. The clinical efficacy of PRP remains under debate, and a standardized protocol has not yet been established [45]. So, physicians should select proper PRP preparations after considering their bio-molecular characteristics and patient indications [46].

Recently, the use of low-level led therapy (LLLT) has been suggested as a treatment for AGA and to improve hair re-growth. LLLT was proposed by the authors 15 days after each treatment to stimulate hair regrowth during the HF-MSCs and PRP treatment and every 3 weeks after the treatment until 6 months post-treatment. Eleven studies were reviewed by Afifi et al. [47]. 9 studies assessing hair count/hair density found statistically significant improvements in both males and females following LLLT treatment. Additionally, hair thickness and tensile strength significantly improved in 2 studies. Patient satisfaction was reported in 5 works.

Autologous platelet-rich plasma (A-PRP), is also now associated with improved surgical outcomes and lower recurrence rates when incorporated in the treatment protocol for the gingival recession and keloid therapies, respectively [48,49].

In dermatological use, differences were found when PRP therapies were performed with the delivery of activated autologous PRP (AA-PRP) in place of non-activated A-PRP. When A-PRP is used with autologous thrombin to yield AA-PRP, it is possible to observe the healing of chronic wounds and shortened recovery times for deep burns [50,51]. Likewise, laser resurfacing of acne outcomes affords qualitatively better results with fewer side effects when performed in conjunction with either topical or intradermal application of calcium-activated PRP [52]. These results, probably, may be attributed to the release and concentration of alpha-granule proteins, including growth factors and cytokines, that stimulate cellular differentiation and proliferation, angiogenesis, and vascular modeling [53].

In the treatment of hair loss, topical use of AA-PRP to harvested follicles prior to implantation has already been shown to increase their survival rate by 15% [54].

Moreover, patients treated with calcium gluconate-activated PRP exhibit increased hair density three months post-surgery with terminal hair density (diameter > 40 μm) increasing by 19% during that time [16].

These findings were confirmed in a study following AGA patients treated with calcium-activated PRP over the course of one year [55]. Twelve weeks later the last injection of PRP, hair density peaked with a 19% increase over baseline measurements; at the one-year mark, hair density fell to 7% above baseline measurements but this value still constituted a significant increase in hair density compared to the baseline values [55].

The growth factors (GFs) obtained by the degranulation of the alpha-granules have been shown to stimulate hair re-growth. In detail, insulin-like growth factor-1 (IGF-1) stimulates proliferation of cycling Ki67^+^ basal keratinocytes [56,57], while transforming growth factor β1 (TGF-β1) protects the proliferative potential of basal keratinocytes by inhibiting cell growth and terminal differentiation [58,59]. Platelet-derived growth factor AA (PDGF-AA) increase the hair inductive activity of DPCs when applied in combination with fibroblast growth factor 2 (FGF-2) [60,61]. Vascular endothelial growth factor (VEGF) stimulates angiogenesis, and PDGF-BB is a potent chemo-attractant for wound macrophages and fibroblasts and stimulates these cells to release endogenous growth factors, including TGF-β1, that promote new collagen synthesis [62].

DPCs harvested from human scalp have shown increased proliferation, increased Bcl-2 and FGF-7 levels, activated ERK and Akt proteins, and up-regulation of β-catenin when cultured in an activated PRP-supplemented growth medium [63]. Since each of these factors positively influences hair re-growth through cellular proliferation to prolong the anagen phase (FGF-7) [28], inducing cell growth (ERK activation) [64], stimulating hair follicle development (β-catenin) [65], and suppressing apoptotic cues (Bcl-2 release and Akt activation) [66,67]. Human scalp affected by AGA injected with PRP should display marked increases in cellular activity. Indeed, histological examination of A-PRP and AA-PRP treated scalp from our previous works [3,17,18] provides such clinical evidence. In both patient populations, the authors observed improvement in the number of follicular bulge cells and follicles, epidermal thickening, improved vascularization, and a higher number of Ki67^+^ basal keratinocytes in PRP-treated scalp tissue compared with placebo.

## 5. Conclusions

Our information obviously highlights the constructive effects of Micrografts-HF-MSCs and/or PRP infusions on AGA. Micrografts-HF-MSCs and PRP may fill in an effective and a safe procedure alternative to AGA; PRP and/or HF-MSCs represent a promising therapy for AGA. The increment of Wnt signaling in DPCs evidently is the important factor that enhances hair re-growth. DPCs have shown increased Bcl-2 and FGF-7 levels, activated ERK and Akt proteins, and upregulation of β-catenin when cultured in an activated PRP-supplemented growth medium. 

## Figures and Tables

**Table 1:** Study design and clinical results obtained using Micrografts containing HFSCs and autologous platelet-rich plasma not activated (A-PRP).

Platelet-Rich Plasma	Micrografts
Injections performed in three session spaced 30 days	Injections performed in two sessions spaced 60 days
Mechanical and controlled injection	Mechanical and controlled injection
Addition of low-level led therapy (LLLT) 15 days after each treatment and every 3 weeks after the third treatment until 6 months post-treatment	Addition of low-level led therapy (LLLT) 15 days after each treatment and every 3 weeks after the second treatment until 6 months post-treatment
Hair density measurements for A-PRP (not activated-PRP) 12 weeks later the last treatment: 65 ± 5 hairs/cm^2^	Hair density measurements for Micrografts 12 weeks later the last treatment: 39 ± 5 hairs/cm^2^
Hair density improvement for A-PRP (not activated-PRP) 12 weeks, later the last treatment, compared with placebo area: 31 ± 2%	Hair density improvement for Micrografts, 12 weeks later the last treatment compared with placebo area: 30 ± 5.0%
Hair density improvement for A-PRP (not activated-PRP) 23 weeks, later the last treatment, compared with placebo area: 28 ± 2%	Hair density improvement for Micrografts, 23 weeks later the last treatment compared with placebo area: 29 ± 5.0%

**Table 2:** Clinical results obtained using A-PRP versus AA-PRP.

A-PRP (Not Activated)	AA-PRP (Activated)
Hair density measurements for A-PRP, 12 weeks later the last treatment: 65 ± 5 hairs/cm^2^	Hair density measurements for AA-PRP, 12 weeks later the last treatment: 28 ± 4 hair cm^2^
Hair density improvement for A-PRP 12 weeks, later the last treatment, compared with placebo area: 31 ± 2%	Hair density improvement for AA-PRP 12 weeks, later the last treatment, compared with placebo area: 19 ± 3%
Hair density improvement for A-PRP 23 weeks, later the last treatment, compared with placebo area: 28 ± 2%	Hair density improvement for A-PRP 23 weeks, later the last treatment, compared with placebo area: 15 ± 3%
